# uFlowAM: an unsupervised framework for detection and visualization of abnormal intracardiac microflow on early-pregnancy fetal cardiac microflow imaging

**DOI:** 10.3389/fped.2026.1888687

**Published:** 2026-07-09

**Authors:** Yan Xia, Yarui Wei, Zhanru Lan, Ning Wu, Yongxin Li, Xueqin Ji

**Affiliations:** 1 Department of Ultrasound Medicine, Peking University First Hospital Ningxia Women and Children’s Hospital (Ningxia Hui Autonomous Region Maternal and Child Health Hospital), Yinchuan, Ningxia Hui Autonomous Region, China; 2 The Third Clinical College, Ningxia Medical University, Yinchuan, Ningxia, China; 3 Department of Ultrasonography, Ningxia Guyuan Maternal and Child Health Hospital, Guyuan, Ningxia Hui Autonomous Region, China; 4 Department of Thoracic Surgery, Beijing Genertec Aerospace Hospital, Beijing, China; 5 School of Automation and Intelligence, Beijing Jiaotong University, Beijing, China

**Keywords:** anomaly detection, congenital heart disease, early pregnancy, fetal cardiac microflow imaging, fetal cardiology, SlowFlowHD, unsupervised learning, visualization

## Abstract

**Background:**

Congenital heart disease (CHD) is a clinically important fetal anomaly. Early-pregnancy fetal cardiac microflow imaging (FCMI) can show low-velocity intracardiac flow, but brief shunt-related, regurgitant, and outflow-tract disturbances remain difficult to recognize when image quality, fetal position, and gestational age vary across examinations.

**Objective:**

To evaluate uFlowAM for fetus-level detection and visualization of abnormal intracardiac microflow patterns on early-pregnancy FCMI.

**Methods:**

This multicenter diagnostic accuracy study analyzed 650 early-pregnancy FCMI examinations from fetuses referred for suspected CHD or CHD risk assessment, including 500 examinations in the internal cohort and 150 in the external cohort. Standard four-chamber, left ventricular outflow tract (LVOT), and right ventricular outflow tract (RVOT) clips were processed by microflow extraction, cardiac-cycle alignment, signal normalization, and 16-frame windowing. uFlowAM used self-supervised training to learn a 256-dimensional representation of control fetal microflow from temporal-order discrimination and masked-frame reconstruction. Model training used no pixel-level or lesion-level labels. Control embeddings were grouped by view and cardiac phase to build a normal microflow template library, and an abnormality index (AbI) was calculated from latent-space Mahalanobis distances. The operating threshold was calibrated in internal validation and then kept fixed for external testing. Clinical utility was assessed in a 9-reader, 240-case multi-reader multi-case (MRMC) study.

**Results:**

Using the fixed operating threshold (τ* = 2.15), uFlowAM achieved an area under the receiver operating characteristic curve (AUC) of 0.94 (95% CI, 0.92–0.96), sensitivity of 0.92, and specificity of 0.88 in the internal cohort. In the external cohort, AUC was 0.92 (95% CI, 0.88–0.95), with sensitivity of 0.90 and specificity of 0.86. Median reader-level AUC increased from 0.85 to 0.92 with uFlowAM assistance, weighted kappa for subtype agreement increased from 0.62 to 0.78, visibility scores increased from 2.8 ± 0.6 to 4.3 ± 0.5, and median reading time decreased from 78 s to 59 s. Mean inference time was 6.8 ± 1.3 s per case.

**Conclusions:**

In this CHD-enriched referral/risk-assessment cohort, uFlowAM detected abnormal early-pregnancy fetal cardiac microflow patterns and improved reader consistency and efficiency on selected fetal cardiac microflow views. The framework should be considered an assistive second-reader tool for early fetal CHD assessment. It should not be used as a substitute for a complete fetal echocardiographic examination.

## 1 Introduction

Congenital heart disease (CHD) is a major contributor to perinatal and infant morbidity and mortality worldwide (1, 2). In early-pregnancy fetal cardiac assessment, the practical difficulty is not only obtaining standard cardiac views but also recognizing small and transient abnormalities in intracardiac microflow. Findings such as small shunts, regurgitant signals, and outflow-tract disturbances may influence follow-up intensity, referral timing, and perinatal planning when they are confirmed by subsequent assessment.

Early-pregnancy FCMI, including SlowflowHD-based low-velocity flow visualization, can complement grayscale fetal cardiac evaluation by displaying fine intracardiac flow in real time. Interpretation remains dependent on acquisition conditions and reader experience, particularly when a flow disturbance is small, temporally brief, or variably captured across four-chamber and outflow-tract views. Techniques such as 4D-flow cardiovascular magnetic resonance (CMR) and computational fluid dynamics (CFD) provide detailed hemodynamic information in selected settings, but their acquisition, post-processing, and expertise requirements limit routine use in first-trimester fetal cardiac assessment (3–7). This microflow-focused task differs from conventional fetal echocardiographic AI tasks, which usually classify anatomy, segment chambers, or estimate function from grayscale images; here, the target signal is a low-velocity, temporally brief flow deviation that may appear only within a narrow cardiac-phase window.

Many reported cardiovascular artificial intelligence studies have relied on supervised labels for tasks such as chamber segmentation or functional assessment (8). Early-pregnancy fetal CHD assessment poses a different annotation problem: dense labeling of microflow abnormalities is difficult because lesion-level annotations are sparse, the fetal heart is small and motion-prone, and acquisition protocols vary across centers. Unsupervised and self-supervised anomaly-detection methods are therefore relevant to this setting because they can learn control microflow patterns and flag deviations without requiring pixel-level lesion labels (9–12).

We evaluated uFlowAM as a label-efficient framework for modeling early-pregnancy fetal cardiac microflow, deriving a fetus-level AbI, and producing review-only visual overlays. The analysis combined internal and external validation with an MRMC reader study. We hypothesized that phase-aligned representation learning and phase-/view-matched normal templates would improve fetus-level detection of abnormal microflow patterns while leaving final interpretation to the clinician. The framework was therefore designed for selected FCMI views as an assistive second reader and not as a replacement for recommended complete fetal echocardiographic assessment.

## 2 Materials and methods

### 2.1 Study design and population

This multicenter diagnostic accuracy study included pregnant participants carrying early-pregnancy fetuses who underwent FCMI because of suspected CHD or predefined CHD risk assessment. Eligible examinations contained standard fetal four-chamber, LVOT, and RVOT clips when clinically obtainable, with sufficient microflow signal and image quality for preprocessing. Reference standard assignment was based on concordant later-gestation fetal echocardiography, postnatal echocardiography, pregnancy outcome or pathologic confirmation when available, or post-delivery clinical follow-up. Examinations were excluded for severe fetal or maternal motion artifact, incomplete standard-view coverage, nonrecoverable microflow signal clipping or aliasing, or failed fetal cardiac-phase estimation/alignment. Each fetus contributed one index early-pregnancy FCMI examination; when more than one eligible examination was available for the same pregnancy, the earliest examination satisfying standard-view coverage and quality-control criteria was retained, and repeat examinations were excluded from analysis. Twin pregnancies were not included in the analytic cohort, thereby avoiding within-pregnancy clustering. The unit of analysis was therefore one fetus/one index examination.

**Figure 1:**
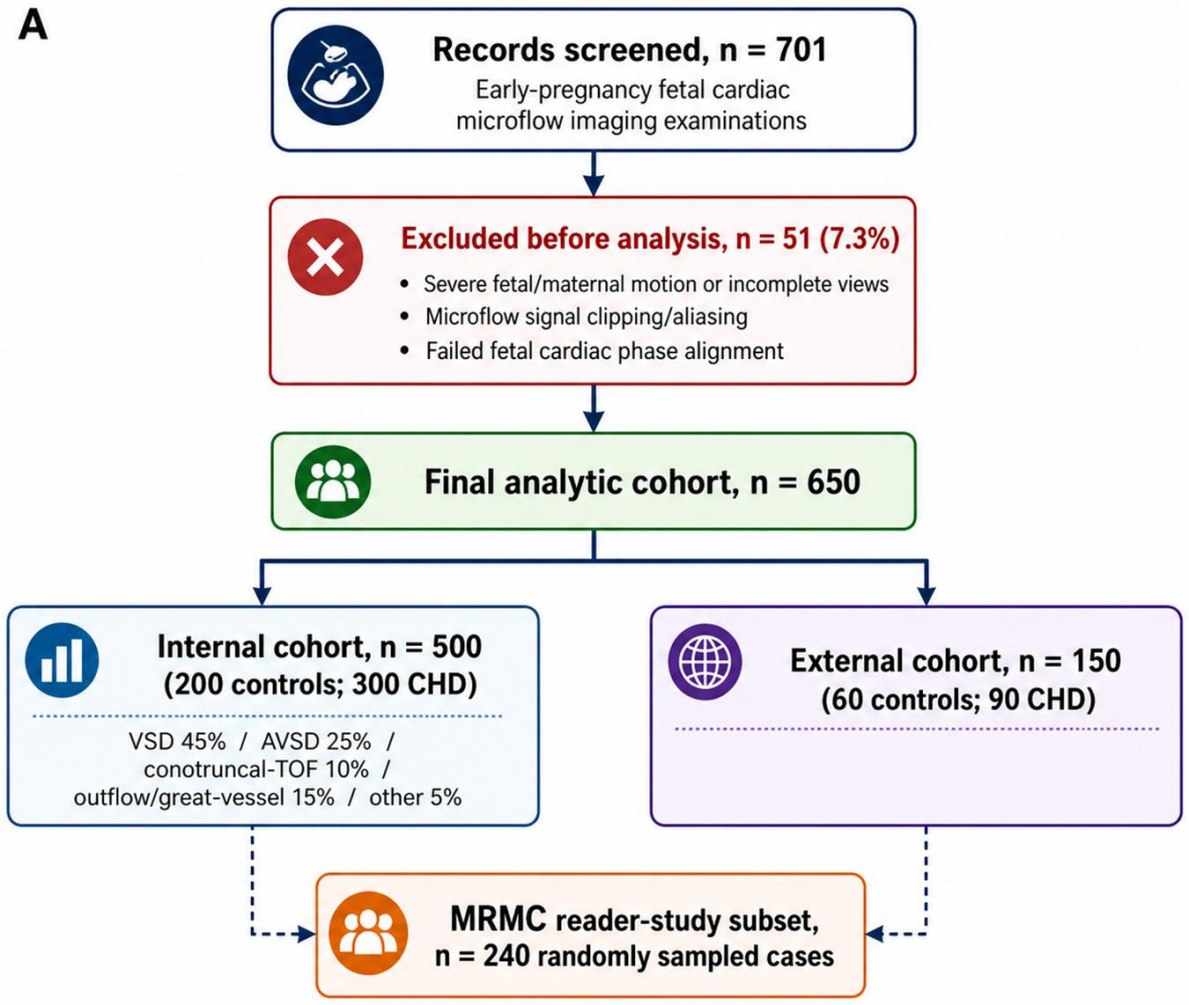
Study flow and cohort composition. Of 701 screened early-pregnancy FCMI examinations, 51 were excluded before analysis. The final analytic cohort comprised 500 internal fetal examinations and 150 external fetal examinations from an enriched suspected-CHD/risk-assessment population. Each fetus contributed one index examination, and twin pregnancies were not included in the analytic cohort.

Of 701 screened examinations, 51 (7.3%) were excluded, leaving 650 examinations for analysis (Figure 1). The cohort was enriched for suspected or confirmed CHD rather than designed as a population-screening sample. The internal cohort included 500 examinations (200 controls and 300 CHD cases), and the external cohort included 150 examinations (60 controls and 90 CHD cases) from three fetal medicine or obstetric ultrasound centers. Within the internal cohort, a stratified fetus-level 70/30 split by center, gestational-age group, and diagnostic category was used for model development and internal validation; all clips from the same fetus/pregnancy remained in the same subset. Median gestational age was 12.3 weeks (interquartile range [IQR], 11.6–13.4 weeks), median crown-rump length was 63 mm (IQR, 52–73 mm), median maternal age was 30.6 years (IQR, 27.4–34.2 years), and median image quality was 4 on a 5-point scale (IQR, 3–4). The internal CHD spectrum included ventricular septal defect (VSD) in 135/300 cases (45.0%), atrioventricular septal defect (AVSD) in 75/300 (25.0%), conotruncal anomalies including tetralogy of Fallot (TOF) in 30/300 (10.0%), outflow-tract/great-vessel abnormalities in 45/300 (15.0%), and other lesions in 15/300 (5.0%). The ‘other lesions’ category comprised rare abnormalities that did not meet the predefined VSD, AVSD, conotruncal/TOF, or outflow-tract/great-vessel categories, including valve abnormalities, venous-system anomalies, and complex or unclassified CHD.

All participating institutions obtained ethics approval, and written informed consent was obtained from pregnant participants and/or their legal representatives as required. Maternal-fetal data were de-identified before analysis. Reporting was aligned with the STARD 2015 and CLAIM recommendations (13, 14).

### 2.2 Image acquisition and preprocessing

FCMI clips were acquired according to local early-pregnancy fetal cardiac protocols at approximately 30 frames per second with clip durations of 5–10 s, using transvaginal or transabdominal probes and low-velocity microflow presets optimized for early gestational cardiac flow. Standard fetal four-chamber, LVOT, and RVOT views were collected whenever clinically feasible. Each DICOM series underwent quality control to verify file integrity, view coverage, and usable microflow dynamic range. Probe types included high-frequency transvaginal endocavitary probes (nominally 5–9 MHz) and transabdominal curved-array probes (nominally 2–9 MHz) in both the internal and external centers; vendor-specific low-velocity microflow presets were retained but were harmonized during preprocessing.

Preprocessing included adaptive microflow extraction from grayscale fetal cardiac anatomy, cardiac-cycle alignment based on image-derived periodicity, spatial resizing to 256 × 256 pixels, normalization of the displayed microflow signal to a common 0–1 range, and temporal segmentation into 16-frame windows. During model development, only mild topology-preserving augmentation was applied, including rotation within ±5 degrees, temporal jitter of ±1 frame, and luminance normalization. Pixel-level and lesion-level labels were not used for model training; fetus-level labels were used only for threshold calibration and performance evaluation.

### 2.3 Unsupervised model development

**Figure 2:**
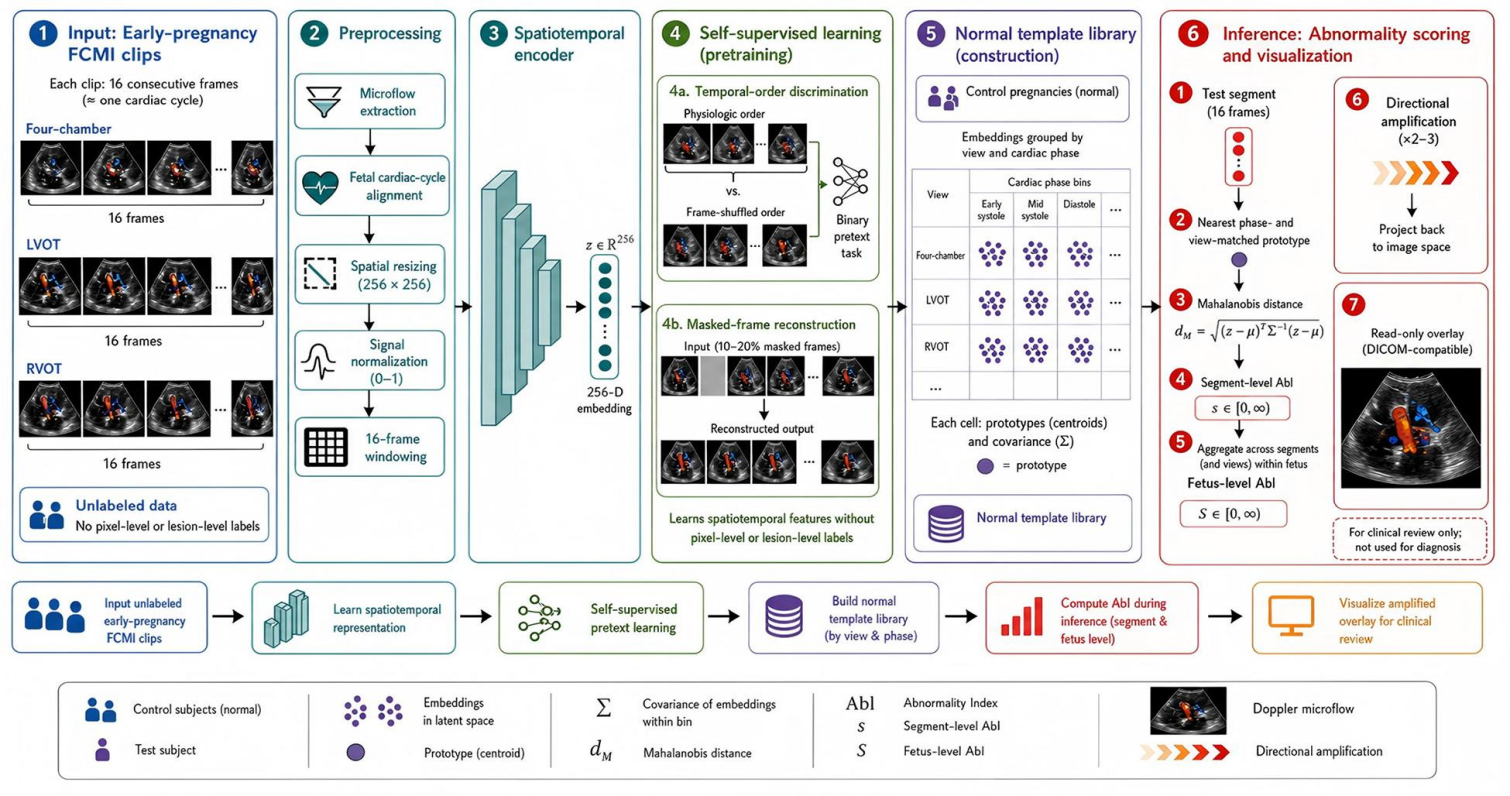
Simplified uFlowAM architecture and inference workflow. Standard FCMI clips are preprocessed into 16-frame segments, encoded into 256-dimensional embeddings by a self-supervised spatiotemporal encoder, matched to phase- and view-specific normal prototypes, and converted into segment-level and fetus-level AbI scores. A read-only DICOM-compatible overlay is generated for clinical review; diagnostic interpretation remains based on the original examination and clinical context.

uFlowAM maps each preprocessed 16-frame FCMI segment to a 256-dimensional latent embedding using a spatiotemporal encoder (Figure 2). The input views were the four-chamber, LVOT, and RVOT clips described above. Training used two self-supervised pretext tasks. Temporal-order discrimination asked the encoder to distinguish physiologic frame order from frame-shuffled sequences, whereas masked-frame reconstruction asked the decoder to reconstruct 10%–20% randomly hidden frames. After training, control embeddings were organized by view and cardiac phase to form the normal template library used for AbI scoring and overlay generation. Briefly, the encoder consisted of four spatiotemporal convolutional residual blocks with 3 × 3 × 3 kernels, normalization, GELU activation, and progressive temporal-spatial downsampling, followed by global spatiotemporal average pooling and a two-layer projection head that generated the 256-dimensional embedding. A lightweight mirrored decoder was used only for masked-frame reconstruction and was not used for AbI scoring at inference.

Let x denote a preprocessed fetal cardiac microflow tensor with dimensions T × H × W (T = 16; H = W = 256). A spatiotemporal encoder f*θ* mapped each segment to a 256-dimensional embedding z = f*θ*(x).

For temporal-order discrimination, a shuffled segment $\tilde{x}$ was generated by random frame permutation. A binary classifier $g_{\phi }$ predicted whether a segment preserved its original temporal order. The corresponding loss was

$$L_{\mathrm{ord}}=-y\log\;\hat{y}-(1-y)\log(1-\hat{y}),$$

where y∈{0,1} denotes the temporal-order label and $\hat{y}=g_{\phi }(z)$.

For masked-frame reconstruction, a binary mask *m* hid 10%–20% of frames, and a decoder $d_{\psi }$ reconstructed the missing content. The reconstruction loss was

$$L_{\mathrm{rec}}=∥(1-m)⊙(x-d_{\psi }(m⊙x)){∥}_{1}.$$

The total training objective was

$$L=\lambda_{\mathrm{ord}}L_{\mathrm{ord}}+\lambda_{\mathrm{rec}}L_{\mathrm{rec}},$$

The two loss terms were equally weighted (both weights set to 1). Training used the Adam optimizer with an initial learning rate of 1 × 10−4, batch size 32, and early stopping based on tuning-set loss.

Training was allowed to continue for up to 100 epochs, and the checkpoint with the lowest validation loss in the development workflow was retained. The model was implemented in PyTorch 2.7 with mixed precision. These settings were chosen to reduce dependence on lesion-level annotation while preserving a compact representation of control microflow dynamics.

### 2.4 Abnormality index and visualization module

Embeddings from control examinations in the development subset were grouped by imaging view and cardiac phase and clustered to form normal microflow prototypes (Figure 2). For each test segment, the nearest phase- and view-matched prototype was identified, and the segment-level AbI was defined as the Mahalanobis distance between the test embedding and that prototype distribution. Fetus-level scoring aggregated segment-level distances across views and temporal windows. The fixed operating threshold (τ* = 2.15) was calibrated in the internal validation set to balance sensitivity and specificity, and the same threshold was used for external evaluation. Prototype construction used only control embeddings from the development subset. Embeddings were stratified by imaging view and normalized cardiac-phase bin; within each stratum, k-means centroids served as candidate prototypes, sparse strata were represented by a single centroid, and a minimum cluster size was enforced before covariance estimation. The covariance matrix for each prototype was estimated from the assigned control embeddings using shrinkage with diagonal loading before inversion, and the same stored centroids, covariance matrices, and threshold were used unchanged for internal validation and external testing.

For visualization, the latent residual component associated with the abnormality score was amplified approximately 2- to 3-fold and projected back to image space as a read-only overlay. The underlying FCMI frame was not modified. The overlay was intended to direct attention to candidate abnormal flow regions during review, not to serve as an independent diagnostic decision. For a fixed preprocessed input, trained checkpoint, normal-template library, and operating threshold, overlay generation was deterministic. However, scan-rescan reproducibility across separately acquired clips was not assessed. Because lesion-level or pixel-level ground truth was unavailable, overlays were interpreted only as qualitative attention maps and were not used to claim localization of known anatomic lesions.

### 2.5 Reader study

A 9-reader MRMC study assessed clinical utility in a randomized subset of 240 examinations with an approximate CHD-to-control ratio of 3:2. Readers comprised three junior, three intermediate, and three senior physicians experienced in fetal cardiac or obstetric ultrasound. Each case was reviewed under two conditions, separated by a washout interval of at least 2 weeks: unaided FCMI review and FCMI review with the uFlowAM score and overlay. Readers were blinded to the reference standard. Case randomization was performed at the fetus/examination level using a computer-generated random sequence stratified by cohort source, CHD/control status, and major CHD subtype so that the reader-study subset approximately preserved the distribution of the analytic cohort. The order of aided and unaided reading sessions was counterbalanced across readers, and readers reviewed cases after a washout interval of at least 2 weeks.

Primary reader-study endpoints were fetus-level AUC, sensitivity, and specificity. Secondary endpoints included weighted kappa for fetal CHD subtype agreement, per-case reading time, and abnormality visibility scores rated independently on a 5-point scale by a separate panel of five senior experts.

### 2.6 Statistical analysis

Continuous variables are summarized as mean ± SD or median (IQR), and categorical variables are summarized as counts or percentages. The primary study endpoint was fetus-level AUC for detection of abnormal early-pregnancy fetal cardiac microflow patterns consistent with CHD. Sensitivity, specificity, positive predictive value (PPV), negative predictive value (NPV), and F1 score were reported at the fixed operating threshold. Because the analytic cohort was enriched for CHD, PPV and NPV were interpreted only in the context of the study prevalence. AbI distributions between controls and CHD cases were compared using the Mann–Whitney U test. Reader aided-versus-unaided performance was compared at the paired reader level; reading time was analyzed with paired nonparametric testing and visibility with paired parametric testing as appropriate. Two-sided *P*values < 0.05 were considered statistically significant.

Deployment feasibility was evaluated using ONNX/TensorRT on a single RTX 4080 GPU. Mean processing time was 6.8 ± 1.3 s per fetal examination, and the 90th percentile was below 10 s.

Exploratory baseline and ablation experiments were performed on the same internal validation split to assess the contribution of individual design components. Baseline comparators included a convolutional reconstruction autoencoder, an f-AnoGAN-style latent/reconstruction anomaly score, and a self-supervised embedding with global Mahalanobis scoring. Ablation variants removed cardiac-cycle phase alignment, replaced phase-/view-matched templates with global control templates, removed temporal-order discrimination, removed masked-frame reconstruction, or replaced Mahalanobis AbI with Euclidean distance. All variants used the same preprocessing, fetus-level split, threshold-calibration rule, and outcome definitions; external testing was not repeated for these exploratory comparisons.

## 3 Results

### 3.1 Cohort composition and data quality

Of 701 screened early-pregnancy FCMI examinations, 51 (7.3%) were excluded before analysis, leaving 650 fetal examinations in the final dataset. The final analytic cohort included 500 internal fetal examinations and 150 external fetal examinations. Exclusions reflected severe fetal or maternal motion artifact, incomplete standard fetal cardiac views, nonrecoverable microflow signal clipping or aliasing, and failed fetal cardiac phase alignment after preprocessing (Table 1). The final dataset contained one index examination per fetus/pregnancy, and no twin pregnancies were included.

**Table 1:** Study cohort composition and imaging characteristics.

Characteristic	Value
Records screened	701
Excluded before analysis	51 (7.3%)
Final analytic cohort	650
Internal cohort	500 (200 controls; 300 CHD)
External cohort	150 (60 controls; 90 CHD)
MRMC reader-study subset	240 randomly sampled cases
Gestational age, weeks	12.3 (11.6–13.4)
Crown-rump length, mm	63 (52–73)
Maternal age, years	30.6 (27.4–34.2)
Image quality score (1–5)	4 (3–4)
Data completeness	Exceeded 90% for all predefined quality fields (successful fetal cardiac phase estimation, standard-view coverage, and usable microflow dynamic range)
Internal CHD spectrum	VSD 135/300 (45.0%); AVSD 75/300 (25.0%); conotruncal/TOF 30/300 (10.0%); outflow-tract/great-vessel abnormality 45/300 (15.0%); other 15/300 (5.0%); other category included rare valve abnormalities, venous-system anomalies, and complex/unclassified CHD
Unit of analysis	One index early-pregnancy FCMI examination per fetus/pregnancy
Repeat examinations from the same pregnancy	Not included; one eligible index examination was retained when repeat examinations were available
Twin pregnancies	Not included in the analytic cohort

Data are *n* (%), median (IQR), or as otherwise stated. The cohort was enriched for suspected or confirmed CHD; the internal CHD spectrum refers to the 300 CHD cases in the internal cohort. Each fetus contributed one index examination. The ‘other’ category included rare valve abnormalities, venous-system anomalies, and complex/unclassified CHD.

The median image quality score was 4/5 (IQR, 3–4). Data completeness for successful fetal cardiac phase estimation, standard-view coverage, and usable microflow dynamic range exceeded 90% in the final analytic cohort.

### 3.2 Primary diagnostic performance

**Figure 3:**
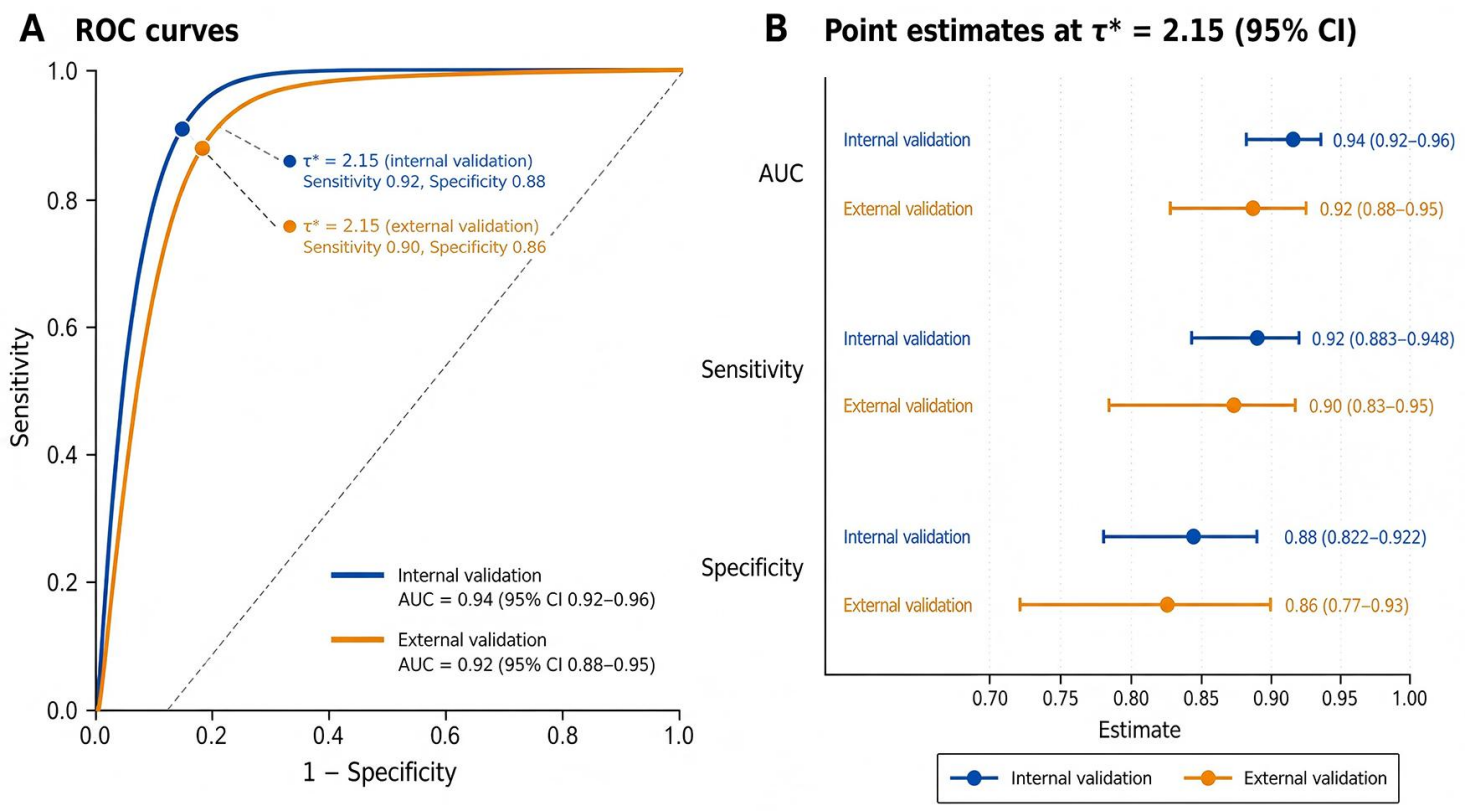
Cohort-level diagnostic performance of uFlowAM. **(A)** Receiver operating characteristic curves based on fetus-level AbI scores in the internal and external cohorts. **(B)** Point estimates and 95% confidence intervals for area under the curve, sensitivity, and specificity at the fixed operating threshold (τ* = 2.15).

At the fixed operating threshold (τ* = 2.15), uFlowAM achieved an internal AUC of 0.94 (95% CI, 0.92–0.96), sensitivity of 0.92 (95% CI, 0.883–0.948), and specificity of 0.88 (95% CI, 0.822–0.922). In the external multicenter cohort, the corresponding values were AUC 0.92 (95% CI, 0.88–0.95), sensitivity 0.90 (95% CI, 0.83–0.95), and specificity 0.86 (95% CI, 0.77–0.93). Figure 3 and Table 2 summarize cohort-level performance.

**Table 2:** Primary diagnostic performance of uFlowAM.

Cohort	AUC (95% CI)	Sensitivity (95% CI)	Specificity (95% CI)	PPV	NPV	F1
Internal validation	0.94 (0.92–0.96)	0.92 (0.883–0.948)	0.88 (0.822–0.922)	0.92	0.88	0.92
External validation	0.92 (0.88–0.95)	0.90 (0.83–0.95)	0.86 (0.77–0.93)	0.91	0.85	0.90

PPV and NPV are prevalence-dependent and are specific to the CHD-enriched study cohort (60% CHD); they should not be interpreted as first-trimester population-screening values.

At the CHD-enriched study prevalence of 60%, PPV and NPV were 0.92 and 0.88 internally and 0.91 and 0.85 externally. These prevalence-dependent values should not be extrapolated to first-trimester population screening. Mean inference time was below 10 s in 90% of cases, indicating computational feasibility for an assistive review workflow.

Exploratory baseline and ablation analyses are summarized in Table 3. The full uFlowAM configuration achieved the highest AUC (0.94). Reconstruction-based baselines produced lower AUCs (0.86–0.87), and a self-supervised embedding with global Mahalanobis scoring achieved an AUC of 0.89. Among uFlowAM ablations, removal of cardiac-cycle alignment (AUC 0.90) or phase-/view-specific template matching (AUC 0.91) produced larger decreases than removing a single pretext task or replacing Mahalanobis AbI with Euclidean distance. These results suggest that the phase-aligned, view-/phase-matched template design contributed most to performance, whereas the self-supervised tasks and AbI design provided additional incremental benefit; because the analysis was limited to the internal validation split, it should be considered exploratory.

**Table 3:** Exploratory internal-validation baseline and ablation analyses.

Configuration	AUC (95% CI)	Sensitivity	Specificity
Full uFlowAM	0.94 (0.92–0.96)	0.92	0.88
Convolutional reconstruction autoencoder	0.86 (0.82–0.90)	0.83	0.78
f-AnoGAN-style latent/reconstruction baseline	0.87 (0.83–0.91)	0.84	0.80
Self-supervised global Mahalanobis baseline	0.89 (0.85–0.92)	0.86	0.82
No cardiac-cycle phase alignment	0.90 (0.86–0.93)	0.88	0.82
Global template instead of phase/view templates	0.91 (0.87–0.94)	0.88	0.84
No temporal-order discrimination	0.92 (0.89–0.95)	0.90	0.86
No masked-frame reconstruction	0.91 (0.88–0.94)	0.89	0.85
Euclidean AbI instead of Mahalanobis AbI	0.92 (0.89–0.95)	0.90	0.86

All exploratory estimates used the same internal validation split, preprocessing, fetus-level outcome definition, and threshold-selection rule as the main analysis. External validation was not repeated for these variants.

### 3.3 Abnormality index separation

**Figure 4:**
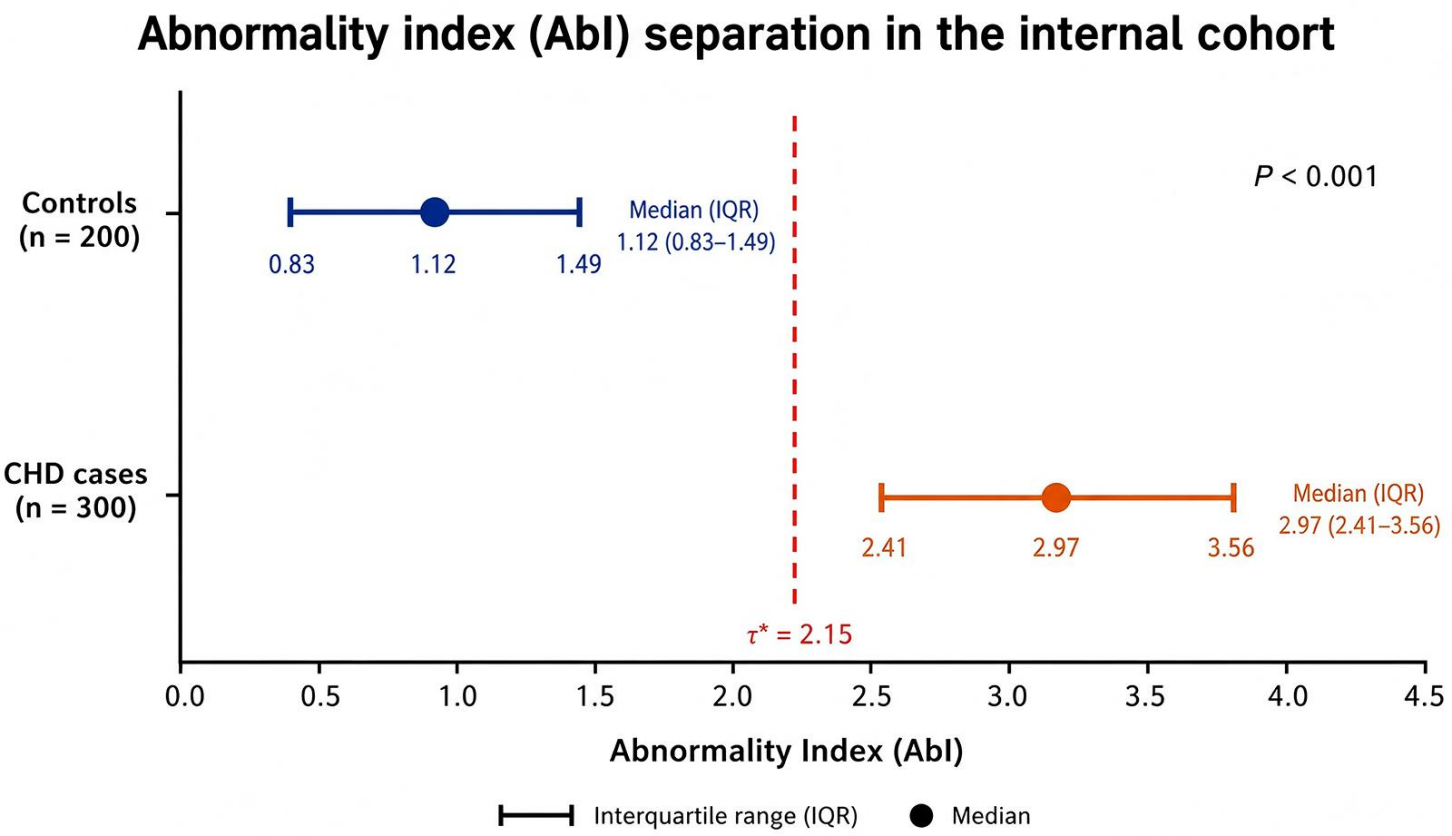
Abi distribution in the internal cohort. Horizontal intervals show interquartile ranges and circles show medians. The dashed vertical line marks the fixed operating threshold (τ* = 2.15).

AbI values differed between controls and fetal CHD cases in the internal cohort. Median (IQR) AbI was 1.12 (0.83–1.49) in controls and 2.97 (2.41–3.56) in CHD cases (*P* < 0.001). The fixed threshold of 2.15 fell between the median values of the two groups (Figure 4), consistent with the intended use of AbI as a summary measure of deviation from control microflow templates.

### 3.4 Subgroup analysis

Subgroup analyses were exploratory because several lesion categories contained limited numbers of cases. AUC estimates were numerically high across the major CHD subtypes represented in the internal cohort, including VSD, AVSD, conotruncal anomalies including TOF, outflow-tract/great-vessel abnormalities, and other lesions (Table 4). The estimate for VSD was 0.95. Estimates for conotruncal/TOF cases and the heterogeneous other category should be interpreted cautiously because of limited sample size. The other category was retained as a single exploratory group because individual valve, venous-system, and complex/unclassified lesions were too infrequent for separate performance estimation.

**Table 4:** Exploratory subgroup performance by CHD subtype in the internal cohort.

Subtype	AUC (95% CI)	Sensitivity	Specificity
VSD (*n* = 135)	0.95 (0.92–0.97)	0.94	0.88
AVSD (*n* = 75)	0.93 (0.89–0.96)	0.91	0.87
Conotruncal anomaly/TOF (*n* = 30)	0.91 (0.84–0.96)	0.88	0.86
Outflow-tract/great-vessel abnormality (*n* = 45)	0.92 (0.87–0.96)	0.90	0.88
Other (*n* = 15); rare valve/venous/complex lesions	0.90 (0.79–0.97)	0.87	0.86

The “other” category was heterogeneous and included rare valve abnormalities, venous-system anomalies, and complex/unclassified CHD; subtype-specific performance for these individual lesions was not estimated because of small case counts.

### 3.5 Reader study and workflow impact

**Figure 5:**
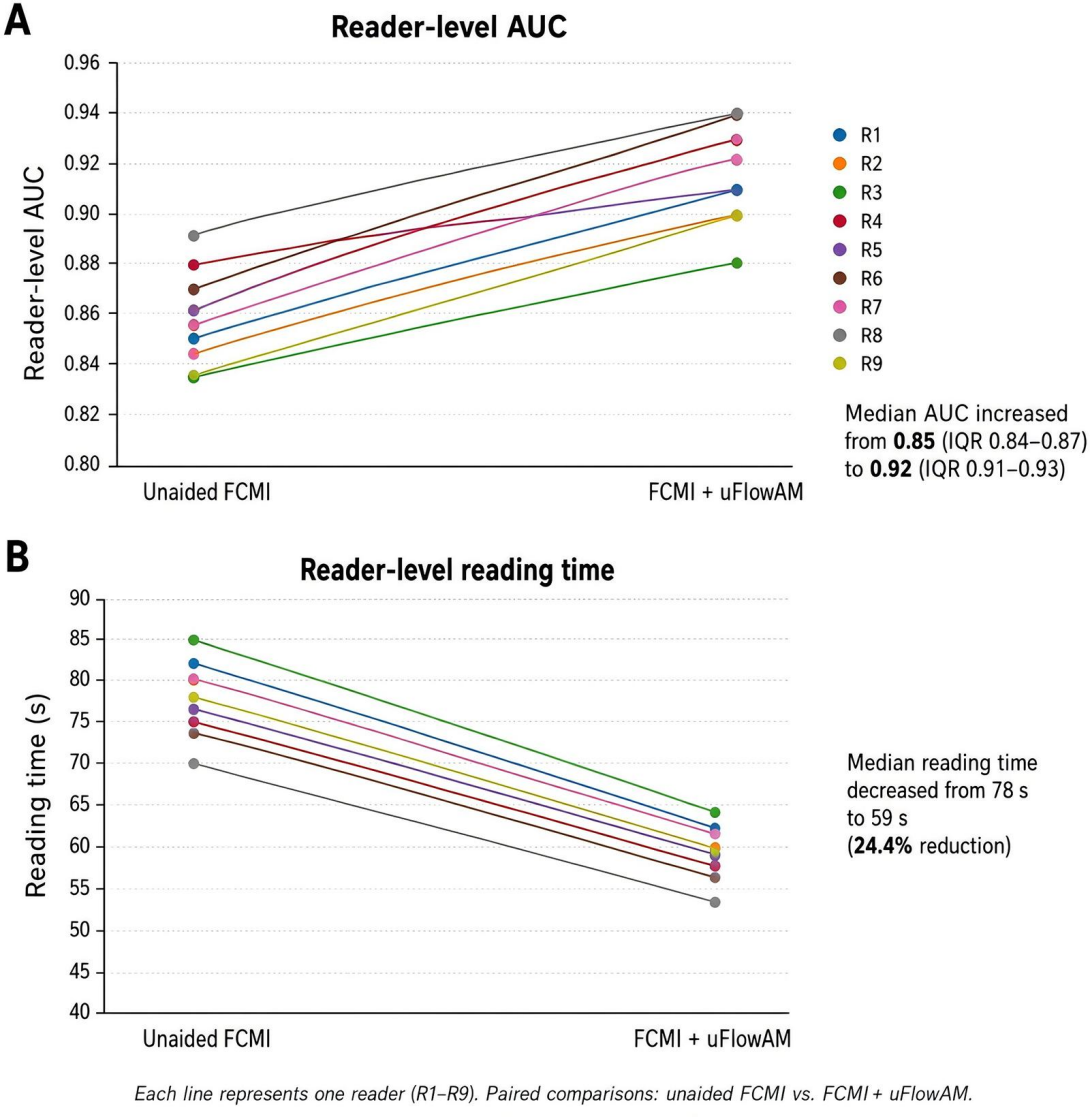
Reader-study analysis of uFlowAM assistance. **(A)** Paired reader-level AUC with and without uFlowAM support. **(B)** Paired reader-level reading time with and without uFlowAM support. Median reader-level AUC increased from 0.85 to 0.92, while median reading time decreased from 78 s to 59 s.

In the MRMC experiment, reader-level AUC increased after uFlowAM assistance for all nine readers. Median reader-level AUC changed from 0.85 (IQR, 0.84–0.87) during unaided review to 0.92 (IQR, 0.91–0.93) with assistance, with per-reader gains ranging from +0.05 to +0.08 (Figure 5 and Table 5). Median reading time changed from 78 s to 59 s per fetal examination, corresponding to a 24.4% reduction.

**Table 5:** Descriptive reader-level performance and Reading time with and without uFlowAM assistance.

Reader	Unaided AUC	Aided AUC	Delta AUC	Unaided time (s)	Aided time (s)	Time reduction (%)
R1	0.84	0.91	+0.07	82	62	24.4
R2	0.85	0.91	+0.06	80	60	25.0
R3	0.83	0.88	+0.05	85	64	24.7
R4	0.88	0.93	+0.05	75	57	24.0
R5	0.86	0.92	+0.06	77	58	24.7
R6	0.87	0.94	+0.07	74	56	24.3
R7	0.85	0.93	+0.08	81	61	24.7
R8	0.89	0.94	+0.05	70	53	24.3
R9	0.84	0.90	+0.06	78	59	24.4

Time reduction was calculated as (unaided - aided)/unaided   ×   100. Across readers, weighted kappa increased from 0.62 to 0.78 and visibility scores improved from 2.8 ± 0.6 to 4.3 ± 0.5.

Weighted kappa for fetal CHD subtype agreement increased from 0.62 to 0.78 (95% CI for the difference, +0.11 to +0.21), and visibility scores increased from 2.8 ± 0.6 to 4.3 ± 0.5. In this reader experiment, the score and overlay were associated with higher agreement, higher visibility ratings, and shorter reading time.

### 3.6 Qualitative error analysis

**Figure 6:**
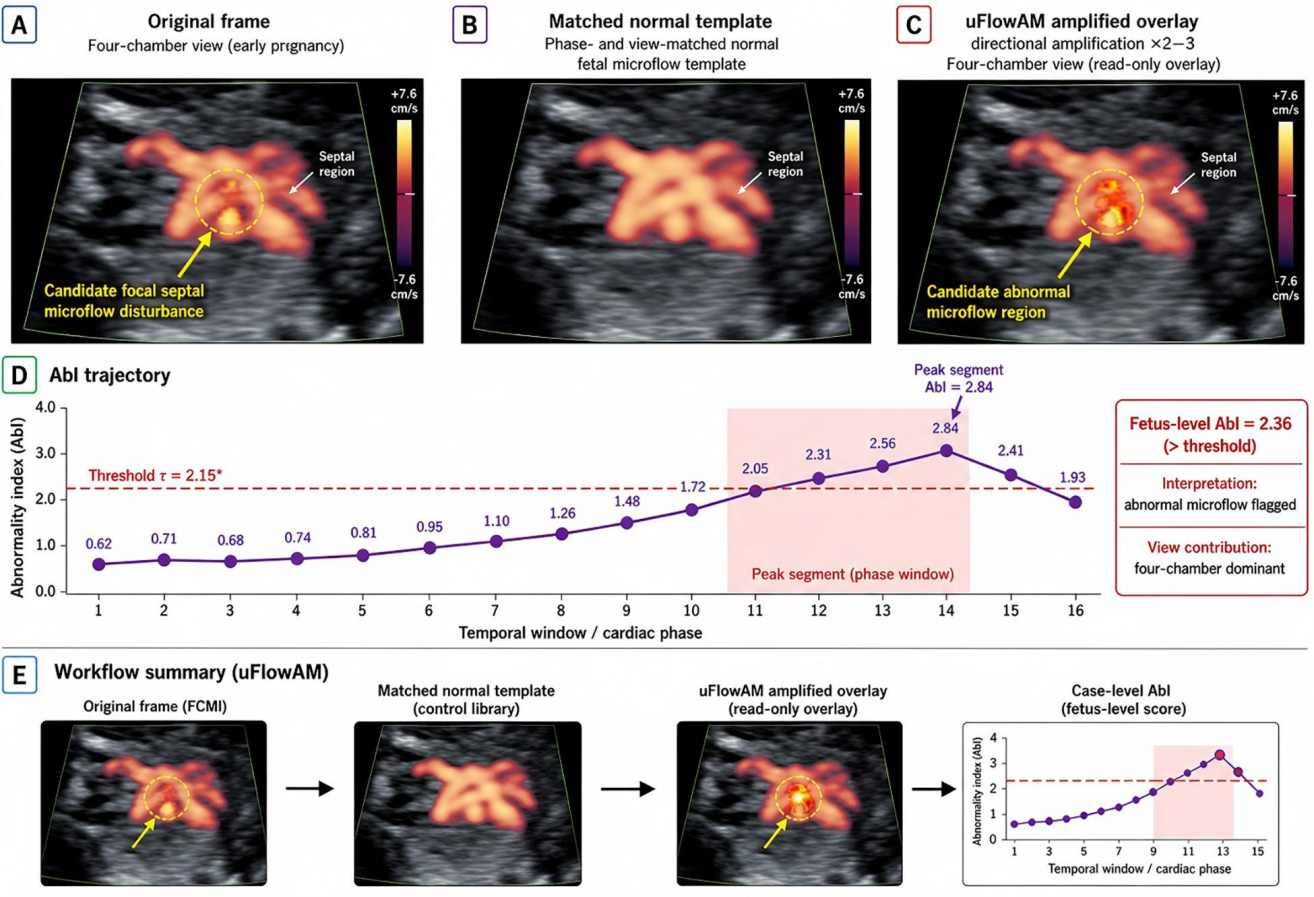
Representative case-level visualization produced by uFlowAM. **(A)** Original four-chamber early-pregnancy FCMI sector frame showing a focal septal jet-like microflow disturbance. **(B)** Phase- and view-matched normal fetal microflow template selected from the control library. **(C)** Read-only overlay generated by directional amplification (approximately 2- to 3-fold), highlighting the candidate abnormal microflow region while preserving the underlying fetal FCMI frame. **(D)** AbI trajectory across 16 temporal windows/cardiac-phase bins. The peak segment AbI (2.84) and fetus-level AbI (2.36) exceed the fixed operating threshold (τ* = 2.15), indicating that abnormal fetal cardiac microflow was flagged in this case. **(E)** Workflow summary from the original frame to the matched template, amplified overlay, and case-level score. The overlay is a review aid and should not be interpreted as lesion-level ground truth. AbI, abnormality index; FCMI, fetal cardiac microflow imaging.

Among discordant cases reviewed qualitatively, false-positive examinations were usually related to valve-related aliasing, off-axis fetal cardiac acquisition, residual fetal or maternal motion artifact, or local microflow blooming. False-negative examinations tended to involve very small or intermittently captured jets or suboptimal fetal cardiac phase alignment. Because this analysis was qualitative, these patterns should be interpreted as failure-mode observations rather than formal estimates of error prevalence. Figure 6 provides a representative case-level visualization in which uFlowAM compares the original four-chamber FCMI frame with a phase-matched normal fetal microflow template, generates a read-only amplified overlay over the focal septal jet-like region, and displays the AbI trajectory across temporal windows. In the displayed case, the peak segment AbI was 2.84 and the fetus-level AbI was 2.36, both exceeding the fixed operating threshold (τ* = 2.15); this output is intended to guide attention rather than establish a stand-alone diagnosis. These patterns are reported as qualitative failure examples to avoid overinterpreting the overlay.

## 4 Discussion

### 4.1 Principal findings

In this multicenter diagnostic accuracy study of selected early-pregnancy FCMI views, uFlowAM separated control from CHD examinations with high fetus-level AUCs in internal and external cohorts. In the MRMC experiment, assistance with the score and overlay was associated with higher reader AUC, agreement, visibility ratings, and shorter reading time. The primary contribution of the framework is an assistive second-reader approach for flagging atypical microflow patterns, rather than autonomous diagnosis. The algorithmic contribution is the phase-aligned, self-supervised control-template scoring workflow, whereas the clinical contribution is its integration into a second-reader workflow for selected early-pregnancy FCMI views. The study should therefore be interpreted as a feasibility and assistive diagnostic-accuracy evaluation, not as proof that uFlowAM is superior to all alternative AI architectures.

### 4.2 Relation to prior work

Supervised labels have supported many cardiovascular AI studies, including echocardiographic functional assessment (8). Early-pregnancy fetal microflow analysis presents a different annotation problem: dense lesion-level labels are difficult to obtain and may be unreliable when flow abnormalities are transient, small, or view dependent. uFlowAM addresses this constraint by learning a control microflow representation and scoring deviations from phase- and view-matched templates. This approach is consistent with broader unsupervised anomaly-detection work in medical imaging (9–11) and label-efficient representation learning in cardiovascular data (12, 15). Unlike supervised fetal CHD classifiers or lightweight real-time diagnostic networks (16), uFlowAM does not learn CHD labels directly; instead, it estimates deviations from control microflow dynamics.

Advanced flow-characterization methods such as 4D-flow CMR and CFD remain important for comprehensive hemodynamic assessment in selected later-gestation or postnatal settings (3–7). However, their cost, acquisition burden, and post-processing requirements limit routine use in early-pregnancy fetal cardiac assessment. The intended role of uFlowAM is narrower: to augment available FCMI clips during review and identify segments that may merit closer inspection, rather than to replace comprehensive hemodynamic assessment.

### 4.3 Clinical implications and interpretability

Clinically, the findings suggest that an AbI score and review-only overlay may help readers identify subtle or fleeting microflow deviations in early-pregnancy examinations. The overlay should not be treated as a pathophysiologic explanation or as pixel-level lesion localization. The original FCMI clip must remain available, and interpretation should incorporate standard fetal echocardiographic findings, obstetric ultrasound findings, and clinical context. uFlowAM should be used only as an adjunct to recommended fetal cardiac imaging views and comprehensive fetal echocardiographic assessment. A low AbI on the three selected FCMI views should not be interpreted as a negative complete fetal echocardiogram when clinical suspicion remains.

### 4.4 Limitations

Several limitations merit emphasis. First, the study focused on selected early-pregnancy fetal cardiac views rather than full fetal cardiac examinations; lesions not represented in four-chamber, LVOT, or RVOT clips may be underdetected. Second, the analytic cohort was enriched for suspected or confirmed CHD, so prevalence-dependent metrics such as PPV and NPV should not be extrapolated to population screening. Third, although the external cohort was multicenter, the sample size was modest, and some subtype estimates were based on small case counts. Fourth, lesion-level ground truth was not available for validating overlay localization. Fifth, the baseline and ablation experiments were exploratory, used a single internal validation split, and were not repeated in the external cohort. Finally, prospective workflow validation is required before clinical deployment. In addition, only one index examination per fetus/pregnancy was analyzed and twin pregnancies were excluded, so longitudinal repeat-examination and multifetal-pregnancy performance remain unknown. The relative contributions inferred from the internal baseline and ablation comparisons should therefore be considered hypothesis-generating rather than definitive. Overlay scan-rescan reproducibility and lesion-level localization accuracy also require prospective validation.

### 4.5 Future directions

Future studies should prioritize prospective multicenter testing in routine early-pregnancy FCMI workflows, predefined comparisons with established anomaly-detection baselines, and vendor-aware calibration across microflow presets. Linking AbI values with lesion severity, later-gestation confirmation, postnatal outcomes, and management decisions would clarify the clinical meaning of the score. Future prospective studies should include routine full fetal echocardiographic views, predefined external comparisons with representative unsupervised and self-supervised baselines, confirmatory ablation analyses, scan-rescan assessment of overlay reproducibility, and lesion-level adjudication when feasible.

## 5 Conclusions

uFlowAM provides an unsupervised approach for detection and visualization of abnormal intracardiac microflow on selected early-pregnancy FCMI views. In this CHD-enriched multicenter cohort, it achieved high internal and external diagnostic performance and was associated with improved reader consistency and efficiency in an MRMC experiment. The findings support further prospective evaluation of uFlowAM as an assistive second-reader framework. uFlowAM should not be used as a stand-alone substitute for complete fetal echocardiography, and prospective validation with prespecified baselines is required before clinical deployment.

## Data Availability

The raw data supporting the conclusions of this article will be made available by the authors, without undue reservation.
